# Selected science: an industry campaign to undermine an OSHA hexavalent chromium standard

**DOI:** 10.1186/1476-069X-5-5

**Published:** 2006-02-23

**Authors:** David Michaels, Celeste Monforton, Peter Lurie

**Affiliations:** 1 The Project on Scientific Knowledge and Public Policy, Department of Environmental and Occupational Health, The George Washington University School of Public Health and Health Services, 2100 M Street NW, Suite 203, Washington, DC, 20037, USA; 2 Public Citizen Health Research Group, 1600 20^*th *^Street NW, Washington, DC, 20009, USA

## Abstract

While exposure to hexavalent chromium (Cr(VI)) has been associated with increased lung cancer risk for more than 50 years, the chemical is not currently regulated by the U.S. Occupational Safety and Health Administration (OSHA) on the basis of its carcinogenicity. The agency was petitioned in 1993 and sued in 1997 and 2002 to lower the workplace Cr(VI) exposure limit, resulting in a court order to issue a final standard by February 2006. Faced with the threat of stronger regulation, the chromium industry initiated an effort to challenge the scientific evidence supporting a more protective standard. This effort included the use of "product defense" consultants to conduct *post hoc *analyses of a publicly-funded study to challenge results viewed unfavorably by the industry.

The industry also commissioned a study of the mortality experience of workers at four low-exposure chromium plants, but did not make the results available to OSHA in a timely manner, despite multiple agency requests for precisely these sorts of data. The commissioned study found a statistically significant elevation in lung cancer risk among Cr(VI)-exposed workers at levels far below the current standard. This finding changed when the multi-plant cohort was divided into two statistically underpowered components and then published separately. The findings of the first paper published have been used by the chromium industry to attempt to slow OSHA's standard setting process. The second paper was withheld from OSHA until it was accepted for publication in a scientific journal, after the rulemaking record had closed.

Studies funded by private sponsors that seek to influence public regulatory proceedings should be subject to the same access and reporting provisions as those applied to publicly funded science. Parties in regulatory proceedings should be required to disclose whether the studies were performed by researchers who had the right to present their findings without the sponsor's consent or influence, and to certify that all relevant data have been submitted to the public record, whether published or not.

## Background

In recent years, efforts by major corporations to deflect unwanted scientific findings have been reported increasingly in the lay and biomedical literature. The tobacco industry, for example, used the attorney-client privilege to shelter scientific studies from disclosure [1-3]; it also funded apparently independent organizations to provide a patina of credibility for its work [1,4]. Pharmaceutical manufacturers have withheld unfavorable clinical trial results [5,6] and have disparaged research that produced unwelcome findings [7,8].

We report a case in the less-scrutinized field of occupational health in which all these elements were combined in a coordinated strategy to challenge the Occupational Safety and Health Administration's (OSHA) action to reduce workers' exposures to the lung carcinogen hexavalent chromium (Cr(VI)).

Cr(VI) is not a newly-identified hazard; the increased risk of lung cancer has been documented in Cr(VI)-exposed workers for more than 50 years [9,10]. Thomas F. Mancuso and Wilhelm C. Hueper, for example, studied the mortality experience of chromium-exposed workers employed between 1931 and 1937 at a Painesville, Ohio facility. Results of their study were published in 1951 [10,11], with updates on the cohorts published by Dr. Mancuso in 1975 [12] and again in 1997 [13], consistently finding an excess risk of lung cancer among exposed workers. Cr(VI) has been classified as a human carcinogen by the National Toxicology Program [14] and the International Agency for Research on Cancer [15].  It is used in chrome plating and in the production of metal alloys and pigments. OSHA estimates that approximately 380,000 U.S. workers are currently exposed to Cr(VI) [16].

At present, OSHA does not regulate Cr(VI) on the basis of its carcinogenicity. The agency's current Permissible Exposure Limit (PEL) of 52 *u*g/m^3 ^was originally recommended in 1943 by the American National Standards Institute as a level adequate to prevent nasal perforations in chromium-exposed workers [17]. This 52 *u*g/m^3 ^limit was adopted by OSHA in 1971 when the agency was created, without any formal review. In 1976, OSHA announced plans to lower the Cr(VI) standard [18] and in 1994, the OSHA administrator acknowledged that "there is clear evidence that exposure...at the current PEL...can result in an excess risk of lung cancer" [19]. However, until recently, no change was officially proposed and the 52 *u*g/m^3 ^PEL remains in effect today.

In 1993, Public Citizen and the Oil, Chemical and Atomic Workers International Union (OCAW) (now part of the United Steelworkers) petitioned OSHA to reduce its PEL from the current level of 52 *u*g/m^3 ^to 0.25 *u*g/m^3^, measured as an 8-hour time-weighted average. Two lawsuits ensued, challenging OSHA's "unreasonable delay" in promulgating a stronger standard. Although the chromium industry, through its trade association the Chrome Coalition, had intervened in the lawsuits on OSHA's behalf opposing a change in the PEL, on April 2, 2003, the U.S. Court of Appeals for the Third Circuit ordered the agency to issue a final rule reducing occupational exposure to Cr(VI) by January 18, 2006 [20], later extended to February 28, 2006. In the words of Judge Edward Becker, OSHA's decade-long delay in issuing a Cr(VI) standard "exceeded the bounds of reasonableness" [21].

### The industry strategy to forestall OSHA rulemaking

Long before the court ruling, however, the chromium industry had initiated an effort to challenge the scientific evidence supporting any stronger OSHA standard, engaging the services of ChemRisk and Exponent, Inc., two consulting firms that specialize in "litigation support" and "product defense" [22,23]. One industry document noted that "this route [hiring the consultants] is expensive and success is not guaranteed, [but] the longer we wait the more difficult the task becomes." [See additional file 1: File1 to view this document.]

In a meeting with chromium industry representatives in 1996, ChemRisk scientists outlined a strategy that included obtaining and analyzing the raw data from a not-yet-published study of Cr(VI) exposure funded by the Environmental Protection Agency (EPA) in order "to forestall the [OSHA] rulemaking." [See additional file 2: File2 to view this document.] Simultaneously, the industry commissioned new publications that questioned the health effects of low levels of exposure to Cr(VI) [24-26], a central issue in any OSHA regulatory initiative. The industry paid for services provided by ChemRisk and Exponent, Inc. through its trade association's attorneys. This arrangement was selected to "...preserve the confidentiality of information, opinion, and data to the extent provided for under the attorney-client privilege and attorney work product privilege." [See additional file 3: File3 to view this document], ensuring that material developed through the process could be sequestered from public view. [See additional file 4: File4 to view meeting summary and plan to preserve attorney-client privilege.]

The industry also contracted with a third consulting firm, ENVIRON [27], to study workers who had only been employed in facilities that were either designed with or converted to production processes that resulted in lower levels of Cr(VI) exposure. ENVIRON was hired through a contract with the Industrial Health Foundation (IHF), a descendant of the Air Hygiene Foundation, an organization founded in 1935 in the wake of the 1930's Gauley Bridge occupational silicosis tragedy to provide employers with confidential assessments of industrial hazards [28]. The study protocol entailed combining workers from four plants using newer, lower-exposure processes – two in the U.S. (Castle Hayne, NC, and Corpus Christi, TX) and two in Germany – into a single cohort. ENVIRON's proposal noted that "the relatively small study sizes and short follow-up periods resulted in a limited ability of [previous] studies to clarify the relationship between modern [low-level] occupational chromate exposures and cancer in general, and respiratory cancers in particular." According to the proposal, creating a single cohort with workers from multiple plants was crucial "to improve statistical power and the inferential value of the results" [29].

In August 2000, the EPA study was published. It is the largest, most comprehensive study ever conducted on the effects of workplace Cr(VI) exposure. The study examined a cohort of more than 2,300 workers employed at a chromate production facility in Baltimore, MD, from 1950 to 1974, and followed through 1992. Exposure histories were reconstructed utilizing 70,000 measures of airborne Cr(VI) concentrations; smoking histories for 93% of the cohort were also incorporated into the analyses. Using OSHA's standard assumption of a 45-year working lifetime, the study reported a significantly elevated lung cancer risk of 1.57 among workers whose mean exposure was at levels just above the PEL requested in the Public Citizen-OCAW petition [30].

No sooner had the EPA study been published than the industry-sponsored critiques began. Scientists with the product defense firm Exponent, Inc. created and analyzed the mortality experience of a "simulated cohort" derived by computer from the EPA study's summary data, standard deviations and ranges [31]. In another report, the consultants obtained the raw data from the EPA study through a Freedom of Information Act request and re-analyzed them [32]. Each of these reports challenged the validity of the EPA study's conclusions and was either entered into the record in litigation or submitted to OSHA by the chromium industry, although not published in the peer-reviewed literature. After extensive analysis, most of the issues raised in these critiques were rejected by OSHA [33].

### OSHA publishes its proposed rule

On October 4, 2004, OSHA published its court-mandated proposed rule for Cr(VI), including a PEL of 1 *u*g/m^3 ^[34]. The agency issued a general request for additional scientific evidence, along with a specific appeal for epidemiological data about the aforementioned cohort in Castle Hayne, where exposure levels were more representative of the concentrations of airborne Cr(VI) found in workplaces today [35]. A mortality study of this group had been published in 1994 [36] and OSHA asked directly, "Are there updated analyses available for [this cohort]?" In addition, OSHA asked, "Are there other cohorts available to look at low exposures?" [37].

Following a three-month comment period, OSHA held 11 days of public hearings [38], at which OSHA reiterated its request for more data and industry repeatedly criticized OSHA for relying on data from high-exposure cohorts [39-44]. In reviewing the hearing transcript, we found no mention by industry representatives or anyone else of any imminent new epidemiological evidence. The public was given until April 20, 2005, to submit additional data and post-hearing comments.

### Selected science

Just weeks before the close of the comment period, a study reporting on the mortality experience of workers employed at the Castle Hayne and Corpus Christi facilities appeared in the *Journal of Occupational and Environmental Medicine *(*JOEM*) [45]. The article had been submitted to *JOEM *in July 2004 and was accepted for publication that October [46], the same month OSHA proposed its rule and specifically asked for information about the Castle Hayne or other cohorts. The analysis has little statistical power (only three lung cancer deaths) and suffers from short follow-up (fewer than half of the workers in the study were followed for twenty years or more, the minimum length of time needed to begin to detect occupational cancer) [47]. Even though they collected data on Cr(VI) exposure, none is presented in the paper and the small sample size precludes logistic regression. Nonetheless, the authors offer the "preliminary conclusion" that "the absence of an elevated lung cancer risk at this time may be a favorable reflection of the post-change [i.e., lower exposure] environment [45]."

Three trade associations made reference to the study in their post-hearing comments [48-50]. For example, the Specialty Steel Industry of North America stated it had "recently" learned of the study:

[W]hile we have not had any opportunity to examine this study...[it] contains potentially incredibly significant data which would allow the development of a dose response relationship based on actual, experienced exposures, as opposed to the modeled exposures upon which OSHA currently relies to set the PEL. Indisputably, this would be much more relevant and appropriate data upon which to establish a risk-based regulatory limit [48].

The Specialty Steel Industry warned that OSHA's failure to consider these results would be "arbitrary and capricious," a legal term, signaling that failure to address these "new" findings would be grounds for a legal challenge. The Society of the Plastics Industry, Inc. (SPI) remarked on the "potentially great significance" [49] of the new ENVIRON study.

Moreover, the comments by these trade associations confirm that they were privy to unpublished details of the ENVIRON analysis. For example, one wrote:

SPI has learned that in the German plants, excess lung cancer mortality was demonstrated only in the highest exposure group, using chromium exposure estimates based on urinary chromium results. It is possible that the data obtained from the German facilities demonstrates that no increase in risk at any but the highest exposure levels to CrVI [49].

The industry thus succeeded in inserting this hearsay material into the record without ever providing the actual study data.

Intrigued by these developments, we conducted an Internet search, using the terms "Industrial Health Foundation" and "Chrome Coalition." To our surprise, we located a notice for a hearing related to the bankruptcy of IHF. In this proceeding, two chromium industry trade associations asserted that files in the possession of IHF actually belonged to the industry, because the IHF was, according to the petitioner, simply a "third-party administrator of the trade association." [See additional file 5: File5 to view this document.] Using the Public Access to Court Electronic Records system [51], we obtained documents filed with the court, some of which have been quoted in this manuscript. These materials also led us to parties in the bankruptcy proceedings who provided additional documents, including ENVIRON's study protocol and the final report of the combined study of the U.S. and German plants.

That report, submitted by ENVIRON to IHF in September 2002 but never by the industry to OSHA and never published in its entirety, provides strong support for the inadequacy of the current standard, and raises questions about whether the proposed OSHA PEL of 1 *u*g/m^3 ^is adequately protective. The ENVIRON authors found a significantly elevated risk of lung cancer mortality associated with exposure to Cr(VI) in these newer low-exposure facilities (SMR = 1.66, 95% CI = 1.08–2.46, using a combination of German national rates and U.S. state rates for comparison; SMR = 1.37, 95% CI = 0.89–2.03, using German and U.S. state rates). The investigators developed a series of job exposure matrices and utilized air monitoring data from the U.S. plants and urine monitoring from the German plants to estimate the exposure history of each worker. In order to convert the urinary measurements (*u*g/L) into air measurements (*u*g/m^3^), we divided by 0.77, the same conversion factor used by the industry [52], and divided by 45, to convert cumulative exposures into mean annual ones.

Logistic regression analyses of the four-plant cohort found increased risk associated with increased cumulative (or lifetime) exposure to Cr(VI). In one analysis, the lung cancer mortality odds ratio among workers with highest annual exposure (≥ 5.8 *u*g/m^3^) was 20.2 (95% CI = 6.2–65.4), compared to the lowest exposure group (< 1.2 *u*g/m^3^) [53]. For the intermediate exposure group (1.2 *u*g/m^3 ^– < 5.8 *u*g/m^3^), the odds ratio was 4.9 (95% CI = 1.5–16.0), also in comparison to the lowest exposure group [53]. Thus, the intermediate group includes exposure at levels only slightly higher than the 1 *u*g/m^3 ^PEL proposed by OSHA in 2004, and showed elevated lung cancer risk at that level.

The final unpublished four-plant report reiterated the strength of the study design: "This study benefited from the multi-site design that provided a reasonably large cohort of post-change [lower exposure] chromium chemical workers, along with the corresponding increase in statistical power generally lacking in previous studies of post-change cohorts" [52].

The published *JOEM *article, however, reports the mortality experience only of workers at the two U.S. plants studied by the ENVIRON researchers. After submitting the results to their sponsors in 2002, the authors evidently separated the German and U.S. results, despite their repeated emphasis in the protocol on the strength of the combined cohort. Instead of a positive result based on four plants, a negative two-plant study was published. In a response to a letter [54] in the *JOEM*, the authors stated that the German component of the study had not been published because it was rejected by a journal to which it had been submitted, and defended the exclusion of the German data on the ground that different exposure measurements (air vs. urine) were used [55]. This claim is not consistent with the need for large sample size to increase statistical power, as stated in the protocol and the final report. In June 2005, we provided the study protocol [29] and final report of the four-plant study [52] to OSHA [56].

On October 17, 2005, the ENVIRON researchers submitted the German component of the study to OSHA, accompanied by a note saying the paper had been accepted for publication in *JOEM *[57,58]. In this manuscript, the ENVIRON researchers report that "lung cancer risk was elevated only in the highest exposure group (SMR = 2.09 95% CI = 1.08–3.65)" [58].

The authors conducted another logistic regression analysis, but in this new version the estimate of relative risk for workers with high exposure is derived by comparing them to workers in the low and intermediate exposure groups combined. The result of this change is the disappearance of the statistically significant increase in lung cancer mortality risk among the intermediate group that was found in the unpublished final report. Tables 1 and 2, adapted from the unpublished final report [52] and the pre-publication manuscript of the German component of the study submitted by the authors to OSHA [58], respectively, compare the results of the two regression analyses. In addition, while the elevation of the lung cancer SMR in the unpublished final report of the four-plant study was statistically significant, when the cohort was divided into two components, the lung cancer SMR was not statistically significant in either the German or U.S. components.

**Table 1:** Elevated lung cancer mortality risk in intermediate and high exposure groups in original unpublished study^†^

Mean Exposure to Cr(VI)*	OR**	95% CI
Low (< 1.2 *u*g/m^3^)	Ref	--
Intermediate (1.2 – <5.8 *u*g/m^3^)	4.9	1.5 – 16.0
High (≥ 5.8 *u*g/m^3^)	20.2	6.2 – 65.4

^†^Adapted from Table 17 in: Final report: Collaborative cohort mortality study of four chromate production facilities, 1958–1998 [53].

*Mean Exposure to Cr(VI) derived by dividing cumulative urinary chromium exposure by 0.77 (conversion factor for air concentration), and then dividing by 45 years (OSHA's working life assumption); see text for details.

**Odds Ratio

**Table 2:** Lung cancer mortality risk in intermediate group disappears after German component of study published separately^††^

Mean Exposure to Cr(VI)*	OR**	95% CI
Low and Intermediate (<5.8 *u*g/m^3^)	Ref	--
High (≥ 5.8 *u*g/m^3^)	6.9	2.6 – 18.2

^††^Adapted from Birk T, Mundt KA, Dell LD, et al: Lung cancer mortality in the German chromate industry, 1958–1998. *J Occup Environ Med *(in press) [58].

*Mean Exposure to Cr(VI) derived by dividing cumulative urinary chromium exposure by 0.77 (conversion factor for air concentration), and then dividing by 45 years (OSHA's working life assumption); see text for details.

**Odds Ratio

## Discussion

Faced with the threat of stronger OSHA regulation of workplace exposure of Cr(VI), a powerful carcinogen, the chromium industry initiated an effort to challenge the scientific evidence that the agency would likely use to justify a new standard. While criticizing OSHA for relying upon data from high-exposure cohorts, the chromium industry also commissioned a study of the mortality experience of workers at four plants with lower exposures, the results of which confirmed the elevated lung cancer risk in such workers. The consultants presented a final report to their chromium industry sponsors in 2002, but industry never provided OSHA a copy of the full four-plant study. Even when the agency specifically asked for precisely these sorts of data during its 2004–2005 rulemaking proceedings, the chromium industry and the authors remained silent.

For publication, industry-funded scientists divided this study into two components and published them separately. The first paper to be published was a statistically underpowered, negative study, the findings of which are being used by industry to attempt to reduce its regulatory burden. The second paper combined two exposure strata from the final report, resulting in the disappearance of the stratum of particular regulatory interest in which a statistically significant finding was apparent in the unpublished final report. This allowed the industry trade associations to make the misleading assertion that elevated lung cancer mortality risk was only seen among workers with the highest exposure histories.

OSHA's statute instructs its decision makers to use the "best available evidence" [59] in the rulemaking process. The circumstances regarding these studies raise troubling questions about the ability of government to effectively issue rules protecting public health when studies are conducted, controlled and selectively published or provided to the rulemaking agency by the regulated industry [8,60]. The entry of the German study into the OSHA record only after it was accepted for publication, months after the regulatory docket closed and years after data collection was complete, raises an important question for public health research: when regulatory proceedings are underway, should potentially important data be sequestered until the peer review process is complete? Many U.S. regulatory agencies, including the EPA and the Food and Drug Administration (FDA) rely heavily on unpublished studies, submitted by study sponsors, in reaching regulatory decisions. In this case, sponsors withheld data that OSHA requested during an active rulemaking process.

It is now widely recognized that pharmaceutical manufacturers have an obligation to report the existence and results of all clinical trials, although this is often not done satisfactorily [61,62]. The higher standards of practice now being sought in the reporting of pharmaceutical trial results should also be applied in occupational health and safety research. The editors of thirteen leading journals will no longer publish articles based on studies done under contracts in which clinical trial investigators did not have the unfettered right to publish the findings, asserting that such restrictions "erode the fabric of intellectual inquiry that has fostered so much high-quality clinical research" [63]. Parties in regulatory proceedings should be required to disclose whether the studies they submit were performed by researchers who had the right to present or publish their findings without the sponsor's consent or influence [64]. Regulatory agencies should weigh the submitted information accordingly.

Public health is not well served by the unequal treatment of public and private science [65]. Parties submitting scientific analyses and reports to the record should be required to disclose the true sponsorship of the study, including the original source of the sponsor's funding. Parties involved in the rulemaking process should also be required to certify that they have submitted all relevant data to the public record, whether or not those data have undergone peer review. Medical journals are increasingly willing to publish findings even if they have already been made available in another form. Regardless, public health rulemakings should not be based on partial records or limited by scientists' career concerns, particularly when lives hang in the balance.

## List of Abbreviations

Cr(VI) Hexavalent Chromium

EPA US Environmental Protection Agency

IHF Industrial Health Foundation

JOEM Journal of Occupational and Environmental Medicine

OCAW Oil, Chemical and Atomic Workers International Union

OSHA US Occupational Safety and Health Administration

PEL Permissible Exposure Limit

SMR Standardized Mortality Ratio

*u*g/m^3 ^micrograms per cubic meter of air

## Competing interests

PL is with Public Citizen's Health Research Group, a party in the lawsuit filed against the US Department of Labor to compel OSHA to issue an occupational hexavalent chromium standard. DM and CM declare that they have no competing interests.

## Authors' contributions

DM, CM and PL researched and wrote the article. All authors read and approved the final manuscript.

## Supplementary Material

Additional File 1 Chrome Coalition Ad Hoc PEL Committee. Summary of Chrome Coalition's meeting with ChemRisk on February 13, 1996. Click here for file

Additional File 2 Chrome Coalition Meeting Minutes. Meeting minutes describing the Chrome Coalition's February 13, 1996 meeting with ChemRisk. Click here for file

Additional File 3 Agreement between Collier, Shannon, Rill & Scott, PLLC, ChemRisk and the Industrial Health Foundation signed September 10, 1996. The agreement outlines services to be provided by ChemRisk on behalf of the Chrome Coalition. Click here for file

Additional File 4 Chrome Coalition Meeting Summary, September 12, 2002. Summary of the Chrome Coalition's meeting on September 12, 2002. Click here for file

Additional File 5 Affidavit of Dr. Joel Barnhart, December 17, 2004, in Re: Industrial Health Foundation, Inc., U.S. Bankruptcy Court for the Western District of Pennsylvania. The affidavit describes, among other things, the Industrial Health Foundation's role with respect to the Chrome Coalition. Click here for file
