# Supplementary material for: Selected science: an industry campaign to undermine an OSHA hexavalent chromium standard
Source: Environ Health. 2006 Feb 23;5:5. doi: 10.1186/1476-069X-5-5 (PMC1402271; doi:10.1186/1476-069X-5-5)
Supplement: Additional File 1 — Chrome Coalition Ad Hoc PEL Committee. Summary of Chrome Coalition's meeting with ChemRisk on February 13, 1996. [file 1476-069X-5-5-S1.pdf]

CHROME COALITION AD HOC PEL COMMITTEE

Special Meeting /Interview with ChemRisk Committee
Discussions and Recommendations

February 13, 1996

BACKGROUND

The driving force for current OSHA regulatory efforts concerning hexavalent chromium, as well as many environmental regulations and toxic tort actions, is the Mancuso study of workers at the Painesville, Ohio, chromium chemicals plant. Although there are many deficiencies in this work, it has been widely accepted by regulatory authorities over the last ten years and it would take a major effort with better data to successfully challenge it.

For the past several years we have expected that the study being conducted at Johns Hopkins University of the former workers at the Baltimore chromium chemicals plant would significantly clarify the relationship between exposure to hexavalent chromium and lung cancer. It was hoped that it would provide a better database than Mancuso. This study was commissioned by EPA and we know that OSHA is planning to use the findings in this study as the basis for setting a new Permissible Exposure Limit (PEL).

We now know that the analysis of this study is much more complicated than originally anticipated since there was limited manipulation of the data. We are concerned about how OSHA will interpret this information. Unless a more complete review of the Johns Hopkins study is conducted we believe that OSHA will maintain their position, that the Chromium PEL should be lowered in the 0.5 to 1.0 ug/m^3 range. The main health concern is the impact of hexavalent chromium on lung cancer.

The Chrome Coalition felt that it was necessary to contract with a well regarded consultant in epidemieology and risk assessment to review all of the information that OSHA might use, determine the limitations and organize and develop a proper scientific basis (model) for predicting the impact of hexavalent chromium on lung

Maxwell
29114

cancer in the workplace. Although this route is expensive and success is not guaranteed, the longer we wait the more difficult the task becomes.

PROPOSALS

Two proposals were received from consultants to specifically address these issues as they relate to the anticipated OSHA proposed standard on workplace exposure to hexavalent chromium. The OSHA/PEL ad hoc committee has reviewed these proposals and after a meeting with representatives of ChemRisk on February 13, 1996 to discuss the proposal and related activities, is recommending that the one submitted by ChemRisk be considered. Dennis Paustenbach of ChemRisk presented his view of the action that should be taken to address all the issues relating to the cancer risk associated with exposure to chromium. He outlined the immediate steps required together with the estimated time requirements and costs as listed below:

1.)	Critically examine the information now in the OSHA docket including the current assessment by ICF Kaiser. Submit relevant information to correct errors and fill deficiencies.	3 - 4 Months	$55,000
2.)	Develop an in-depth analysis of the Mancuso work and publish it in a respected peer review journal.	6 - 8 Months	$40,000
3.)	Publish a detailed manuscript on how the risk associated with exposure to hexavalent chromium should be determined using the most modern techniques or model.	6 - 8 Months	$45,000
4.)	Initiate discussions at as high a level as possiblewithin OSHA to obtain the data associated with the experience of the Baltimore plant workers (Lees Study).	1 - 2 Months	$15,000
5.)	If the Baltimore data is obtained, do a proper analysis correcting for smoking incidence as completely as possible.	18 - 30 Months	$500,000+

Maxwell
29115

As an example of an approach that works, Dennis cited the recent agreement between industry and unions to lower the PEL for 1,3-butadiene from 1000 to 1 ppm. Although OSHA originally wanted to lower it even more, they have announced that they are strongly considering this agreement. The establishment of a PEL for benzene of 0.5 ppm rather than 0.1 ppm was also cited as a recent instance of a large scale effort that produced a result industry could live with that was not as low as OSHA had originally wanted to go.

Dennis Paustenbach discussed several other strategies such as pitting the ACGIH-TLV Committee against the OSHA-PEL Committee by the submission of various information reflecting risk analysis. He also felt very strongly about conducting the analysis and submission of papers that have been peer reviewed into the docket as soon as possible since OSHA would be required to address them in the standard-setting process. And finally, he illustrated the point that the Johns Hopkins data must be thoroughly analyzed beyond what EPA/OSHA had contracted for, so that the issue of hexavalent chromium exposure is evaluated properly now and that further misconceptions like Mancuso are dismissed.

ACTION

I recommend that we begin following the steps outlined above and at each point reevaluate whether to go ahead to the next step or take some other route. As you can see, just going through the first four steps is projected to cost about $120,000 and take us through 1996. American Chrome & Chemicals has verbally committed to one half of this amount pending OxyChem's commitment and whether other members of the Chrome Coalition will contribute. A letter drafted by the OSHA PEL ad hoc committee is being sent out to this effect. The ad hoc committee will recommend contracting with ChemRisk for the steps outlined above at the April 22, 1996 meeting.
