# Supplementary material for: Selected science: an industry campaign to undermine an OSHA hexavalent chromium standard
Source: Environ Health. 2006 Feb 23;5:5. doi: 10.1186/1476-069X-5-5 (PMC1402271; doi:10.1186/1476-069X-5-5)
Supplement: Additional File 2 — Chrome Coalition Meeting Minutes. Meeting minutes describing the Chrome Coalition's February 13, 1996 meeting with ChemRisk. [file 1476-069X-5-5-S2.pdf]

COPY

CHROME COALITION

CHROME COALITION AD HOC PEL COMMITTEE

Special Meeting with ChemRisk

February 13, 1996

Pittsburgh International Airport
Pittsburgh, Pa.

Those in attendance were:

Dr. Donald Billmaier	- AlliedSignal, Inc.
Dr. Joel Barnhart	- American Chrome and Chemicals
Dr. Mark Harris	- Chemical Land Holdings
Dr. Brent Findley	- ChemRisk Division of McLaren/Hart
Dr. Dennis Paustenbach	- ChemRisk Division of McLaren/Hart
Ms Deborah Proctor	- ChemRisk Division of McLaren/Hart
Dr. William Butler	- Environmental Risk Analysis
Mr. Michael Buczynski	- Occidental Chemical Corporation
Ms Stephanie Siegel (via phone)	- Specialty Steel Industry of the U.S.
Mr. James Boldt	- Wayne Pigment Corporation/Color Pigments Manufacturers Association
Ms Marianne Kaschak	- Industrial Health Foundation

The meeting was called to order at 10:05 a.m. by Dr. Joel Barnhart, Chairman of the Chrome Coalition and member of the ad hoc committee.

Ms Kaschak read the Coalition Policy with respect to meetings covered by the antitrust laws. The Chairman emphasized the intent of the Policy and cautioned those present to observe both the letter and spirit of the antitrust laws. Counsel is present, via speaker phone, to insure against infringement. Introductions of the attendees followed.

Dr. Barnhart stated that the purpose of this meeting was to meet with the scientific principals specific to the proposal for critiquing OSHA risk assessment techniques and its standard-setting process relative to a proposed Cr^{+6} PEL. The proposal was jointly prepared by ChemRisk and Environmental Risk Analysis.

Of initial concern was the number of hours allocated for secretarial time for Phase I. Considering all tasks within Phase I, it totaled 136 hours. Dr. Paustenbach justified the secretarial time expenditure, listing the activities of this job function to include assisting with the

Administered by:
INDUSTRIAL HEALTH FOUNDATION, INC. • 34 Penn Circle West • Pittsburgh, Pa. 15206 • (412) 363-6600 • FAX (412) 363-6605

literature search, photocopying, preparing spreadsheets, time sheets, and billing invoices, taking dictation, and acquiring the OSHA docket materials. Responding to an inquiry from Dr. Billmaier, Dr. Butler indicated the secretarial services relative to Environmental Risk Analysis (ERA) would be provided by ChemRisk and are included in the proposal.

Dr. Barnhart asked for an explanation of involvement of the other identified team members. Ms Proctor said that J. Panko, M. Gargas, and T. Long would provide input into industrial hygiene issues and support to the literature reviews. P. Scott will be involved with statistical analyses, and T. Ye will support the risk assessment critique and review.

Addressing Phase II of the proposal, Dr. Barnhart asked that ChemRisk and ERA expand and express their perception regarding scope and accompanying costs. Ms Proctor stated that the latter was contingent on the amount of effort put forth. Dr. Paustenbach concurred and stated that he is quite certain that a positive impact can be made, basically because of the weak database. He then offered a detailed comparison between ChemRisk's activities specific to the benzene rulemaking and similar expected results using the same effort for the Cr^{+6} rulemaking. He further indicated that, during this process, Adam Finkel may be willing to establish a compromise agreement as he had done with 1,3-butadiene. Discussion ensued relative to a threshold concept which was not an issue with benzene.

Dr. Billmaier stated that the Coalition's primary concern in requesting a proposal was to concentrate on the cancer potency. Dr. Paustenbach stated that he has had informal discussions with the principal researchers at Johns Hopkins involved with the chromium epidemiology study. They verified that EPA will not offer additional financial support to complete the study. However, Genevieve Matanowski of Johns Hopkins discussed the possibility of pro bono confidential involvement of ChemRisk to see the study to completion. Dr. Paustenbach suggested that, if this were to come about, the Coalition may wish to approach the regulators with a program designed to fill a "data gap," thus entering into a data gap agreement, to forestall the rulemaking. Ms Siegel indicated skepticism with this tack since the OSHA activity initiated from a union petition and the process is so far along.

In addition to the Mancuso work, ChemRisk/ERA would review and critique all the other relevant epidemiology studies, adding weight to the effort of convincing OSHA not to go forward with what they presently have. This would also insure that there would be enough in the docket to mount a significant legal challenge. As an aside, Ms Proctor said that Dr. Mancuso is about to publish a semi-update of his original study.

In an effort to summarize an appropriate progression of activities, Dr. Billmaier asked Dr. Paustenbach to outline tasks which would provide the Coalition with a concrete base of information to impact the rulemaking and upon which additional future arguments can be made. Dr. Paustenbach said that he would advise completing Phase I and exploring additional work with Peter Lees' data. He concluded by offering the following list of activities and their chronology:

Activity	Proposed Cost
1. Phase I (as described - exclusive of meetings)	$30 K
2. Meetings	?
3. Manuscript describing how to properly do the analysis (dosimetry)	45 K
4. Meeting with Drs. Lees and Matanowski	15 K
5. Developing an anti-Mancuso manuscript	30-45 K

Dr. Barnhart thanked ChemRisk and ERA for answering questions regarding their proposal and for putting the issues into perspective.

After ChemRisk and ERA departed, discussion ensued relative to the comparison of the proposals received from ChemRisk and Ogden Environmental. It was concluded that Ogden will provide information for the record; ChemRisk, on the other hand, affords an opportunity to build for future regulatory impact. Within the next few weeks, Dr. Barnhart will prepare a summary of activities proposed by ChemRisk which will be sent to the ad hoc committee for consideration and approval. In addition, thought must be directed to how and what can be financially supported.

The meeting adjourned.

Respectfully submitted,

Marianne C. Kaschak

Marianne C. Kaschak
Project Coordinator

MCK/sk
