# Supplementary material for: Selected science: an industry campaign to undermine an OSHA hexavalent chromium standard
Source: Environ Health. 2006 Feb 23;5:5. doi: 10.1186/1476-069X-5-5 (PMC1402271; doi:10.1186/1476-069X-5-5)
Supplement: Additional File 3 — Agreement between Collier, Shannon, Rill & Scott, PLLC, ChemRisk and the Industrial Health Foundation signed September 10, 1996. The agreement outlines services to be provided by ChemRisk on behalf of the Chrome Coalition. [file 1476-069X-5-5-S3.pdf]

AGREEMENT

CHROME COALITION

THIS AGREEMENT, dated the 10th day of September 1996, by and between Collier, Shannon, Rill & Scott, PLLC ("CSR&S"), with offices at 3050 K Street, N.W., Washington, D.C. 20007, on behalf of the Chrome Coalition (the "Coalition"), administered by the Industrial Health Foundation, Inc. ("IHF"), a not-for-profit organization of the State of Pennsylvania, with offices at 34 Penn Circle West, Pittsburgh, PA 15206, and The ChemRisk Division of McLaren/Hart Environmental Engineering Corporation ("ChemRisk"), with offices at 8500 Brooktree Road, Suite 300, Wexford, PA 15090, and Environmental Risk Analysis ("ERA"), with offices at 1670 South Amphlett Boulevard, Suite 115, San Mateo, California 94402-2512, (collectively referred to as the "Consultant"), shall be as follows:

1. The term of the Agreement set forth herein shall be for eighteen (18) weeks from the date hereof, unless completed earlier, or unless extended by an amendment to this Agreement, in writing, or unless terminated at an earlier date by either party hereto, by written notice of such intention to terminate mailed to the other party, certified mail, at least fifteen (15) days in advance of the date such termination is to be effective.

2. Collier, Shannon, Rill & Scott, PLLC has been retained by the Coalition to provide advice with respect to the development of a proposed revised Permissible Exposure Limit (PEL) for hexavalent chromium. The purpose of this Agreement is to provide technical information from the Consultant to CSR&S to facilitate the development of legal advice and attorney work product for the Coalition. Accordingly, this Agreement shall be construed in all respects to preserve the confidentiality of information, opinion, and data to the extent provided for under the attorney-client privilege and attorney work product privilege. The Consultant shall perform the

tasks as outlined hereafter, as an independent contractor. This Agreement is not intended to create an association, partnership, agency, joint venture or other relationship of any kind between CSR&S and the Consultant or between the Coalition and the Consultant other than that of an independent contractor. Neither CSR&S nor the Consultant shall have the authority or power to bind the other in any way. The Consultant is employed to render professional services only, and any payments made under this Agreement are compensation for such services rendered.

3. The Consultant is expected to furnish all services reasonably necessary for the full and complete performance of all tasks as described in the Revised Phase I Scope of Work to the Proposal entitled, "Proposal To Provide Expertise In Critiquing OSHA Risk Assessment Techniques And Process For Setting Proposed PEL For Hexavalent Chromium, McLaren/Hart Proposal Number PI96-0011," dated January 31, 1996 ("Revised Proposal"), which is attached hereto as Appendix A. The performance of such tasks shall be referred to as the "Work." The Consultant shall perform the Work in accordance with the schedule set forth in the Revised Phase I Task Schedule and Costs which is attached hereto as Appendix B. The provisions of the Revised Proposal (Appendix A) and the Revised Phase I Task Schedule and Costs (Appendix B) are incorporated in their entirety as if set forth fully herein. The Consultant is not expected to perform, and this Agreement does not include, the tasks described under Phase II of the January 31, 1996 proposal.

4. As full compensation for all services rendered and performed under this Agreement, the Consultant agrees to accept a budget not to exceed $55,775, managed on a time and materials basis. The depth of analysis for each task of the project will be defined by the number of hours approved for each task and will be consistent with Paragraph 17 below.

Payment shall be made as follows: (1) Tasks 1, 2, 4, 5, 6a, and 6b from the Revised Proposal will be billed on completion of each task and delivery of the specified task deliverable; and (2) Task 3 from the Revised Proposal may be billed on a percent completed basis after the first month, with each portion of Task 3 being billed upon completion (up to the projected budget amount). Payment of each invoice shall be due from the Coalition forty-five (45) days from the date the Coalition receives the invoice. Invoices shall not contain project detail (person hours and expenses), but will list the authorized employees who worked on the task in compliance with paragraph 15 below. If this Agreement is terminated, pursuant to Paragraph 1 above, by the Consultant, at any time prior to completion, or if this Agreement is terminated, pursuant to Paragraph 1 above, by CSR&S at any time prior to completion, payment shall be due to the Consultant for the value of services rendered prior to termination. The Consultant will assume responsibility for all its expenses and disbursements in connection with the work described herein, unless the Consultant obtains specific prior written approval from the Coalition for making a disbursement on its behalf for which it will be reimbursed. The Consultant shall furnish a statement to the Coalition setting forth any such amounts, together with supporting material satisfactory to the accountants of the Coalition.

5. CSR&S agrees to review each invoice received from the Consultant promptly (within 10 working days) and, unless an invoice is contested, to forward such invoice to the Coalition for payment under the terms of this paragraph. The Consultant understands and agrees that the Coalition is directly responsible for payment of all invoices submitted under this Agreement and shall not seek to hold CSR&S responsible for such payment. The Consultant is

authorized to communicate directly with IHF, on behalf of the Coalition, on matters pertaining to billing and administration.

6. It is understood by CSR&S that there are some events, such as unexpected or unrelated illness, which are beyond the control of the Consultant and which could result in the necessity to extend the time for completion of the Work described herein. If, in the opinion of the Consultant, such events occur and will extend the time for completion of the Work, the Consultant will promptly inform CSR&S of the reasons for delay and the estimated completion date and submit a written request for extension of time for completion. CSR&S, if such explanation is deemed reasonable, will grant an extension of the time for completion within the limits provided.

7. The Consultant is directed to communicate with and take directions from the Project Coordinator, who will be appointed by CSR&S with approval of the Coalition during this Agreement. The Project Coordinator will assume responsibility for monitoring the project. The Coalition will inform the Consultant by letter as to the identity of the Project Coordinator. Changes in this Agreement must be authorized in writing by the Project Coordinator and any increase in cost must be authorized in writing by CSR&S.

8. The Consultant shall not make any changes in the scope of the Work without written authorization from the Project Coordinator, except that where such changes must be implemented before written authorization is practicable, the required authorization may be given verbally, with a written confirmation following within five (5) working days.

9. The Consultant acknowledges that it does not presently have any agreement or other relationship with another organization, company or governmental entity which would result

in a conflict with the services to be provided in this Agreement, as set forth in Paragraph 3 of this Agreement and otherwise herein, and that it will not enter into any such agreement or contract which would have such a result during the existence of this Agreement, including any future extensions thereof.

10. The Consultant will not disclose to any other person or entity, without the prior written consent of CSR&S and, if the information relates to a specific Coalition member company, the prior written consent of such company, any information concerning the business and other activities of any Coalition member company. CSR&S has the right to review in draft and comment on any documents the Consultant subsequently prepares for publication based in part on work done for CSR&S pursuant to this Agreement. All data, notes, documents, reports and samples that are furnished to the Consultant shall remain the property of CSR&S or the Coalition member company so furnishing such material. This information shall not be published without CSR&S's approval and shall be returned to CSR&S at the conclusion of this Agreement. Similarly, any work product, notes, documents, correspondence, invoices and reports derived from this information shall be the property of CSR&S and shall be forwarded to CSR&S at the termination of this Agreement. These confidentiality obligations shall continue beyond the termination of this Agreement.

11. The Consultant agrees that it shall not publish or disclose any of the results of the Work prior to final completion, and receipt and approval by CSR&S of a final report of the Consultant, to be prepared in accordance herewith, unless specific written approval is granted by CSR&S. CSR&S and the Consultant agree to full public disclosure of scientific information contained in the CSR&S-accepted final report developed through this project. The Consultant

shall advise CSR&S of any scientific information that it deems necessary for inclusion in the OSHA docket. Any scientific information gathered by the Consultant as a result of this Agreement will be given to CSR&S for decision on submission to the docket prior to its submission. CSR&S shall make the final determination as to what information will be submitted to the docket in its name or the name of the Coalition. Any previously published information may be submitted without CSR&S's prior approval.

12. Any reports or work effort provided by the Consultant to CSR&S as services rendered under the Agreement are the property of CSR&S, and shall be forwarded to CSR&S. CSR&S shall have the right to make whatever use is deemed to be appropriate by the Coalition. This provision shall remain binding following the termination of this Agreement.

13. The Consultant, hereby waives and agrees to indemnify, defend, and hold harmless CSR&S and the Coalition from any loss, claim, damage, or liability of any kind arising out of or in connection with this Agreement, except to the extent that such loss, claim, damage, or liability arises in whole from the sole negligence of CSR&S or the Coalition. Liability for losses of any kind shall not exceed $500,000.00.

14. CSR&S and the Coalition hereby waive and agree to indemnify, defend, and hold harmless the Consultant from any loss, claim, damage, or liability of any kind arising out of or in connection with this Agreement, except to the extent that such loss, claim, damage, or liability arises in whole from the sole negligence of the Consultant. Liability for losses of any kind shall not exceed $500,000.00.

15. The Work described in Paragraph 3 of this Agreement, and the other provisions of the Agreement in general, are intended to apply specifically to the Consultant, and not to any

other person or other entity. The ChemRisk/ERA employees who are authorized to work on the project are those described in Section 4.0 of the Proposal dated January 31, 1996. Subcontractors shall be permitted, subject to the review and written approval of CSR&S to the identity and qualifications of each subcontractor and the form of such subcontract. No such approval will relieve the Consultant from any of its obligations under the Agreement. The Consultant agrees to bind each of the Subcontractors to the provisions of this Agreement. The Consultant shall not otherwise delegate or transfer any of its obligations hereunder without the prior written consent of CSR&S. CSR&S will only pay invoices reflecting names of those principals identified in the Proposal or of those whose qualifications have received prior written approval from CSR&S.

16. The Consultant maintains workers' compensation and employer's liability insurance of a form and in an amount as required by the state in which the Work is being performed; other coverages include: commercial general liability (including contractual and products), automobile liability (including owned, non-owned and hired), professional liability, and pollution liability insurance with policy limits of one million dollars ($1,000,000.00). Certificates of insurance to provide evidence of the above coverage will be provided upon request and award of contract.

17. Services performed by the Consultant under this Agreement shall be conducted in a manner consistent with that level of care and skill ordinarily exercised by members of the same profession currently practicing in the same locality under similar conditions. The Consultant warrants that if any of its completed products or services fail to conform to the above professional standard, the Consultant will, at its own expense, perform corrective services of the type originally performed as may be reasonably required to correct such defects, of which the Consultant is notified in writing within six months of the substantial completion of services or

delivery of product. No other representation, express or implied, and no warranty or guarantee is included or intended in this Agreement, or in any report, opinion, document or otherwise.

18. In the event a dispute arises under this Agreement, the Consultant shall notify CSR&S promptly in writing of its contentions and submit its claim together with supporting documentation thereof. If the dispute arises prior to commencement of any one of the identified project tasks, the written notice shall be submitted prior to commencing such task. In any event, the Consultant shall proceed with the Work in compliance with the instructions of CSR&S, but such compliance shall not be a waiver of the Consultant's rights to make a claim, provided it has notified CSR&S in writing as above stipulated.

19. In the event a court of competent jurisdiction holds any provision of this Agreement to be invalid, such holding shall have no effect on the remaining provisions of this Agreement, and they shall continue in full force and effect.

20. This Agreement shall be governed by the laws of the District of Columbia. The Consultant agrees that, in the event of a dispute, the laws of the District of Columbia will apply without regard to the location where the dispute may arise.

21. This Agreement and its enclosures contain the entire agreement between the parties. No amendments or changes to this Agreement shall be effective unless made in writing and signed by authorized representatives of CSR&S and ChemRisk a Division of McLaren/Hart Environmental Engineering Corp.

IN WITNESS WHEREOF, the Consultant and CSR&S have executed this Agreement as of the date first set forth above, to reflect their agreement with the above provisions and to

acknowledge that there are no other understandings or agreements between the parties which are not set forth above.

ChemRisk A Division of
McLaren/Hart Environmental
Engineering Corporation

By: Beverly R. Wehrheim

Name: BEVERLY R. WEHRHEIM

Title: CONTRACTS MANAGER

Collier, Shannon, Rill & Scott, PLLC,
on behalf of the Chrome Coalition

By: [illegible]

John L. Wittenborn

Industrial Health Foundation, Inc.

By: Marianne C. Kaschak

Marianne C. Kaschak

Executive Vice President
