# Supplementary material for: Selected science: an industry campaign to undermine an OSHA hexavalent chromium standard
Source: Environ Health. 2006 Feb 23;5:5. doi: 10.1186/1476-069X-5-5 (PMC1402271; doi:10.1186/1476-069X-5-5)
Supplement: Additional File 4 — Chrome Coalition Meeting Summary, September 12, 2002. Summary of the Chrome Coalition's meeting on September 12, 2002. [file 1476-069X-5-5-S4.pdf]

CHROME COALITION

CHROME COALITION MEETING

September 12, 2002

MEETING SUMMARY

Those in attendance:

Dr. Kenneth Newby	- Atotech GMF Worldwide
Mr. Lawrence Robinson	- Color Pigment Manufacturers Assn.
Mr. Jeff Cox	- Dominion Colour Corporation
Dr. Joel Barnhart	- Elementis Chromium
Mr. Len Etheridge	- Engelhard Corporation
Dr. Mark Harris	- Harris Environmental Risk Management, Inc.
Dr. Nicholas Cory	- Leather Industries of America
Mr. Enrique Castro	- Maxus Energy Inc.
Mr. Russell Morgan	- Occidental Chemical Corporation
Dr. Ross Jones	- Occidental Chemical Corporation
Mr. Joseph Green	- Specialty Steel Industry of NA
Ms. Christina Parascandola	- Specialty Steel Industry of NA
Mr. John Wittenborn	- Specialty Steel Industry of NA
Mr. Jaime Love	- Wayne Pigment Corporation
Ms. Marianne Kaschak	- Industrial Health Foundation

via conference call:

Ms. Robbin Jackson	- Elementis Chromium
Mr. William Allen	- Color Pigment Manufacturers Assn.
Mr. Harold Fitzpatrick	- Color Pigment Manufacturers Assn.

1.0 **Antitrust** policy was read.

1.1 **Introductions** followed and Dr. Jones was welcomed back.

2.0 **Meeting Summary**

2.1 - summary of the May 9, 2002 meeting was accepted as prepared.

3.0 **Financial Report**

- was presented and accepted as distributed.
- funds are critically depleted and given the legal issues pending, decisions for future financing will be considered.

4.0 **Future of the Chrome Coalition**

- producer members may be unable to support at the same level as in the past.

ALL-STATE LEGAL® EXHIBIT 2

- consensus opinion supported the ongoing role of the Coalition as a coordinating body representing all industries.
- driving concern remains the PEL and the advocacy role of the Coalition is paramount to this issue.

Administered by: INDUSTRIAL HEALTH FOUNDATION, INC. • 34 Penn Circle West • Pittsburgh, PA 15206 • (412) 363-6600 • FAX (412) 363-6605

4.0 **Membership Report**

- Coalition presently has 17 members in good standing.
- Since this is the Annual Business Meeting, it was confirmed that a quorum is in attendance.

5.0 **Annual Business Meeting**

5.1 - Officers remain the same for 2003.

Joel Barnhart – Chair
Russell Morgan – Vice Chair

Motion presented by Mr. Robinson; seconded by Mr. Love; passed unanimously.

5.2 - Committee Chairs were appointed and also remain the same for 2003.

John Wittenborn – Clean Air
Nicholas Cory – Solid Waste (pending acceptance of appointment)
Russell Morgan – Clean Water
Mark Harris – Toxicology/Human Effects
Joel Barnhart – International Activities

5.3 - Reciprocal Membership was considered and again approved with ICDA.

Motion to approve was made by Mr. Morgan; seconded by Dr. Newby; passed unopposed.

- Request by ACC's Metal Catalyst Panel to be considered for reciprocal membership was declined.

5.4 - Administration of the Coalition was continued with IHF.

Motion to continue with IHF was made by Dr. Harris; seconded by Mr. Love and passed unopposed.

5.5 - Dues structure will also remain the same for 2003

$12,000 for producer member
1,200 for general member
500 for introductory membership (applicable for two calendar years).

Motion to adopt the dues structure for 2003 was made by Mr. Etheridege, seconded by Mr. Love and

passed unopposed.

6.0 **Standing Committee Reports**

6.1 - Clean Air

- At the end of May, 2002, EPA released its risk characterization results for the 33 air toxics, including chromium. Because of inconsistent reporting, all chromium was amalgamated as hexavalent for dispersion modeling. EPA then based quantitative risk

estimates on the assumption that 34% of the chromium is hexavalent based on past inventorying efforts. The SSINA submitted comments specifically to have EPA re-examine the calculations. The Cr Coalition did likewise. All are encouraged to consider how it impacts their respective industry. Collier Shannon will continue to monitor progress. *Action Item*

6.2 - Solid Waste Committee

- EPA has sent its draft plan for developing the metals assessment framework to the SAB for review. This draft action plan proposes to have SAB's Metals Assessment Panel review the draft metals assessment framework in June 2003 followed by the draft metals guidance in November 2003. SSINA and the Cr Coalition submitted comments. Mr. Green will forward the list of Panel members to IHF for distribution. See attached. *Action Item*

- EPA released its "Priority Chemicals List on September 9, 2002 and of the 30 chemicals listed only 3 metals appeared, cadmium, lead and mercury.

- California's OEHHA recently released its updated Prop 65 status report regarding its NSRLs. The agency is planning to revise the NSRL for Cr^{+6}.

6.3 Clean Water

- On July 24, 2002, NTP presented a design review of the hexavalent chromium drinking water studies. Soon after, the expert scientific panel recommended:
  - including a lower lowest dose suggesting a range of 25 or 10ppm
  - measurement of chromium in target tissues of rats and mice in 2 year study and the special toxicokinetic studies
  - performing blood chemistry analysis in both the above studies
  - not using guinea pigs as test animals

6.4 - Toxicology and Human Effects

- Dr. Barnhart attend the "Second Conference on Molecular Mechanisms of Metal Toxicity and Carcinogenesis held in Morgantown, WV on September 8 – 11, 2002. Thirty five presentations were made and about one-third addressed hexavalent chromium (most at the molecular level). Researchers appear to be concentrating on the reduction of Cr^{+6} to Cr^{+3} outside the cell, mechanisms of the conversion, and the influence of Cr^{+6} on the nucleus. Also, the prevailing opinion was that Cr^{+3} is not carcinogenic; however, there was no indication of any current research on Cr^{+3}.

6.5 - International Activities

- Major concern is End of Life Vehicles, specifically chrome-tanned leather, which both Volkswagen and Audi have ceased using. ICDA continues to follow the issue.

7.0 OSHA PEL Status

- PEL Litigation - court will hear oral arguments in November or early December. Ms. Parascandola indicated that, as an intervenor, the Coalition may be given an opportunity to make oral arguments and she encouraged the Coalition to do so. Collier Shannon will coordinate with CPMA in this effort.
- OSHA Activity
  - there is an assigned a group dedicated to work on the PEL for Cr^{+6}.
  - recent addition to OSHA docket is an economic and technical feasibility study prepared by the Meridian Research group dated December 18, 1994 (entitled "Selected Chapters of an Economic Impact Analysis for a Revised OSHA Standard for Chromium VI: Introduction, Industry Profiles, Exposure Profiles, Technological Feasibility (for 6 Industries) and Environmental Impact"). The Coalition asked Collier Shannon to initiate a FOIA request for the "complete" final report.
  - also recent to the docket is a new OSHA analytical method for Cr^{+6}.
  - request for data, comments and information on all issues relevant to promulgating a standard for Cr^{+6} (Federal Register, 54389, Aug.22, 2002). Comments due by Nov. 20, 2002. Total of 61 questions; Cr Coalition will respond to the first 14 regarding health effects and risk assessment also the last 2 concerning duplication, overlapping and/or conflicting rules.
    - Coalition solicited proposal from Exponent to address the health and risk assessment questions. Consensus supported a revised proposal request eliminating tasks , which appeared redundant or unnecessary.

    - contract with Exponent will be effected through IHF. (This was countermanded during conference calls of 9/20/02 and 10/2/02 when it was decided that the contract be initiated between Collier Shannon and Exponent to insure attorney client privilege).
    - CPMA again stressed coordination of efforts on this issue as well.
    - Collier Shannon will ask OSHA if it will entertain a time extension. *Action Item*
    - formation of an ad hoc committee may be considered to address the OSHA PEL comments.

8.0 Cr Coalition/NIOSH Meeting of 6/13/02 Re: Criteria Document Update

- presented information on Exponent's critique of the Gibb, et al. Publications.
- discussed Painesville cohort.
- lack of coordination between NIOSH and OSHA analytical methods was discussed by Mr. Morgan referencing the work done by Occidental.

- NIOSH asked for written submissions of these presentations. The sponsors of the Painesville work will submit this presentation and IHF will forward the others to NIOSH. Dr. Barnhart will supply IHF with the contact information. *Action Item*

9.0 **Other Matters**

- finances for 2003 may be revisited in a few months with the possibility of a special assessment request

10.0 **Future Meetings**

- the next meeting is scheduled for 9:00 a.m. Thursday, February 6, 2003 at the offices of Collier Shannon, Washington, DC.

- the following meeting is tentatively scheduled for Thursday, May 22, 2003.

- A conference call to discuss a revised Exponent proposal is scheduled for Friday, September 20, 2002 at 12:00 noon EDT. IHF will arrange for a call in number.

Marianne C. Kaschak
Project Coordinator

10/9/02
