# Supplementary material for: Selected science: an industry campaign to undermine an OSHA hexavalent chromium standard
Source: Environ Health. 2006 Feb 23;5:5. doi: 10.1186/1476-069X-5-5 (PMC1402271; doi:10.1186/1476-069X-5-5)
Supplement: Additional File 5 — Affidavit of Dr. Joel Barnhart, December 17, 2004, in Re: Industrial Health Foundation, Inc., U.S. Bankruptcy Court for the Western District of Pennsylvania. The affidavit describes, among other things, the Industrial Health Foundation's role with respect to the Chrome Coalition. [file 1476-069X-5-5-S5.pdf]

IN THE UNITED STATES BANKRUPTCY COURT
FOR THE WESTERN DISTRICT OF PENNSYLVANIA

**In Re:**	)	
	)	
**INDUSTRIAL HEALTH FOUNDATION, INC.,**	)	**CASE NO. 03-25328-BM**
	)	**CHAPTER 7**
**Debtor.**	)	

AFFIDAVIT OF DR. JOEL BARNHART

I, Joel Barnhart, make this declaration based upon my personal experience and knowledge of the events described herein, and state as follows:

1. I am the Vice President of Elementis Chromium.

2. Elementis Chromium is a member of both the Industrial Health Foundation Chromium Chemicals Health and Environmental Committee (the "Chromium Committee") and the Chrome Coalition.

3. I served as one of Elementis Chromium's representatives to both the Chromium Committee and the Chrome Coalition. I therefore have personal knowledge of the relationship between IHF and the two trade associations, and of the business records of the two trade organizations.

4. Additionally, I served as chair of the Chrome Coalition from its inception until the present, with the exception of a few months during that period. *See, e.g.,* Chrome Coalition Meeting Summary dated September 27, 2001 (the "2001 Summary"), Section 4.1, attached hereto as Exhibit "1;" Chrome Coalition Meeting Summary dated September 12, 2002 (the "2002 Summary"), Section 5.1, attached hereto as Exhibit "2." The Chrome Coalition was formed in 1986. *See* Chrome Coalition By-laws and Charter (the "Chrome Coalition By-laws"),

ALL-STATE LEGAL® EXHIBIT A

attached hereto as Exhibit "3" (containing Charter dated 8/1/86). Another Elementis Chromium employee, Gene Renzaglia, was the last named chair of the Chromium Committee.

5. The Chromium Committee and the Chrome Coalition are trade associations organized for the purposes of, among other things: (a) serving as information clearinghouses that gather and disseminate chromium information, research, and studies, especially regarding the health, environmental, and safety aspects of chromium compounds; (b) serving as a facilitator for the chromium industry to respond to proposed regulatory efforts affecting the chromium industry and to recommend and/or participate in litigation affecting the chromium industry; and (c) promoting the chromium industry through distributing information regarding its benefits and positive contributions. *See* Chrome Coalition By-laws (Exhibit 3); Industrial Health Foundation Chromium Chemicals Health and Environmental Committee By-laws (the "Chromium Committee By-laws"), attached hereto as Exhibit "4."

6. Both the Chromium Committee and the Chrome Coalition separately hired IHF to serve as third-party administrator of the trade association. *See* Chrome Coalition By-laws, Article V (Exhibit 3); Chromium Committee By-laws, Article III, Section 4 (Exhibit 4); 2001 Summary, Section 4.4 (Exhibit 1); 2002 Summary, Section 5.4 (Exhibit 2). IHF was not and has never been a member of either the Chromium Committee or the Chrome Coalition. *See, e.g.*, Chrome Coalition By-laws, Article V, Section 2 (Exhibit 3); Chromium Committee By-laws, Article III, Sections 1 and 4 (Exhibit 4).

7. IHF was an agent of the trade associations, and IHF's actions as administrator for the trade associations were subject to the associations' control. *See, e.g.*, Chrome Coalition By-laws, Article V, Sections 2 and 3 (Exhibit 3); Chromium Committee By-laws, Article III, Section 4 (Exhibit 4).

8. As administrator, IHF was the custodian of, among other things, the business records of each of the trade associations, including the minutes from the trade associations' meetings and any correspondence. *See* Chrome Coalition By-laws, Article V, Section 3 (Exhibit 3); Chromium Committee By-laws, Article III, Section 4 (Exhibit 4). The business records of both trade associations were stored by IHF in three file cabinets labeled "Chromium Committee" or "Chrome Coalition" and are now in the Trustee's possession. IHF never owned the business records of the Chromium Committee or the Chrome Coalition, and always was obligated to use the records only in accordance with the associations' directions and for their benefit, and to surrender the records at the associations' demand.

9. On June 24, 2004, I visited IHF and inspected the three file cabinets labeled "Chromium Committee" or "Chrome Coalition" that are currently in the Trustee's possession. These cabinets contain the business records of the Chromium Committee and Chrome Coalition.

I declare, under penalty of perjury under the laws of the United States, that the foregoing is true and correct. Executed on this 16th day of December 2004, in Nueces County, Texas.

Joel Barnhart

Joel Barnhart

STATE OF TEXAS :
: SS
COUNTY OF NUECES :

On the 17th day of Dec, 2004, before me, a notary public, the undersigned, personally appeared Joel Barnhart, known to me to be the person whose name is subscribed to the foregoing instrument and acknowledged to me that he executed the same for purposes and consideration therein expressed.

In witness whereof, I hereunto set my hand and official seal.

(Seal)

Diane LaRoe

Notary Public

DIANE LAROE
MY COMMISSION EXPIRES
April 27, 2008

My Commission Expires:
